# Clinical superiority of HBV RNA testing in patients with chronic hepatitis B

**DOI:** 10.3389/fimmu.2026.1689229

**Published:** 2026-02-25

**Authors:** Jing Zhou, Xiaoyan Li, Yulin Yang, Xiaomin Wang, Wenhua Xu

**Affiliations:** 1 Institute of Regenerative Medicine and Laboratory Technology Innovation, Qingdao University, Qingdao, Shandong, China; 2 Jining Public Health Medical Center, Jining, Shandong, China

**Keywords:** chronic hepatitis B, HBeAg, HBsAg, HBV DNA, serum HBV RNA

## Abstract

**Purpose:**

This study aimed to thoroughly investigate the detection levels of serum Hepatitis B Virus RNA (HBV RNA) and its clinical significance in Chronic Hepatitis B (CHB) patients before and after treatment.

**Methods:**

Serum samples were collected from 487 chronic hepatitis B patients. First, the positivity rate and levels of serum HBV RNA were compared with other relevant indicators, and correlations were examined between untreated and treated CHB patients. Second, treated CHB patients were grouped based on their HBsAg, HBeAg, and HBV DNA status. Differences and correlations between HBV RNA and relevant indicators were further analyzed among the groups.

**Results:**

Among the 487 patients, the positivity rate of HBV RNA was significantly higher than that of both HBV DNA and HBeAg. In the untreated group, the positivity rates of both HBV RNA and HBV DNA were significantly higher than that of HBeAg. In patients who were treated but not cured, the positivity rate of HBV RNA was significantly higher than that of both HBV DNA and HBeAg, regardless of whether patients were grouped based on HBsAg, HBeAg, or HBV DNA levels. When analyzed at specific monitoring levels, patients in the treated group showed significantly lower levels of serum HBV RNA, HBV DNA, HBeAg, HBsAg, ALT, and AST compared to those in the untreated group. Correlation analysis revealed that in untreated patients, HBV RNA was positively correlated with ALT, AST, HBsAg, HBeAg, and HBV DNA. However, in the treated group and in those with low HBsAg, HBeAg, and HBV DNA levels, the correlation between HBV RNA and other markers was weakened.

**Conclusion:**

HBV RNA exhibits greater sensitivity and accuracy in reflecting the status of viruses, particularly in patients undergoing antiviral therapy. It provides unique advantages in monitoring treatment efficacy and assessing persistent viral transcriptional activity.

## 1 Introduction

Chronic Hepatitis B (CHB) is a chronic inflammatory disease of the liver caused by persistentinfection with Hepatitis B Virus (*Orthohepadnavirus hominoidei*, HBV)(1). According to the World Health Organization (WHO), the global seroprevalence of Hepatitis B Surface Antigen (HBsAg) in 2019 was 3.8%. Approximately 296 million people are chronically infected with Hepatitis B virus (HBV), and 820,000 individuals die each year due to complications such as liver failure, cirrhosis, or Hepatocellular Carcinoma (HCC) (2). In China, with the advancement of hepatitis B immunoprophylaxis measures, the prevalence of HBsAg has been decreasing year by year, but due to the large base of CHB patients, there are still about 75 million HBsAg-positive people, and HBV infection is still the main cause of liver failure, cirrhosis, and hepatocellular carcinoma-related deaths (3).

After HBV enters the liver cells, it forms covalently closed circular DNA (cccDNA) under the action of host enzymes. cccDNA serves as a template for viral transcription, which is transcribed into mRNAs with different lengths, and further translates the viral proteins, while some of these mRNAs serve as a template for reverse transcription Formation of a new HBV genome and completion of viral replication (4, 5). In addition, the HBV genome can be integrated into the host hepatocyte genome by host enzymes, but the integrated genome lacks the promoters and enhancers of the core proteins and is unable to undergo viral replication; however, there is a promoter for HBsAg, which results in the sustained expression of HBsAg, and this is the reason why the hepatitis B e antigen (HBeAg) is expressed consistently in HB-negative patients. This is the reason why HBeAg negative patients are HBsAg positive (6, 7).

In the clinical treatment of CHB, first-line nucleos(t)ide analogs (NAs), such as tenofovir disoproxil fumarate (TDF) and entecavir (ETV), have been demonstrated to effectively inhibit HBV DNA replication, significantly reduce viral load, and improve liver tissue lesions with long-term use (8–10). However, the clearance rate of HBsAg is still low and there is the problem of relapse after drug discontinuation (11). The persistence of cccDNA has recently been identified as a major cause of chronicity and relapse after discontinuation of medication in hepatitis B patients (12, 13). However, cccDNA testing requires liver puncture biopsy, which is invasive and potentially infectious, limiting its widespread clinical use (1). Routine clinical HBV DNA testing primarily targets viral DNA in the blood and generally cannot directly detect cccDNA. Therefore, exploring non-invasive and reliable biomarkers that can replace cccDNA to assess intrahepatic hepatitis B virus activity has become a hot topic in CHB research. Traditional serum HBsAg, HBeAg, and HBV DNA are currently the more widely used clinical markers for hepatitis B. However, although quantitative HBsAg assay can assess the infectious load, it is unable to differentiate between integrated DNA and cccDNA sources. While HBeAg and HBV DNA can reflect the replicative activity of viruses in the liver during the natural course of the disease as well as assess the evaluation of the effectiveness of antiviral therapy, they cannot accurately reflect the transcriptional activity of cccDNA either (14).

Serum HBV pregenomic RNA (pgRNA, here described as HBV RNA) is directly derived from cccDNA active transcription in the nucleus of hepatocytes and could theoretically be used to assess cccDNA transcriptional activity. Previous studies have confirmed the correlation between serum HBV RNA water and intrahepatocyte cccDNA levels. Wang et al. demonstrated that serum HBV pgRNA levels, both before and after 48 weeks of peg-IFN therapy, closely correlated with intrahepatic cccDNA levels detected via liver biopsy, reflecting the ongoing transcriptional activity of cccDNA during treatment. It may assist in differentiating the natural history of HBV infection, and the monitoring of serum HBV RNA may provide an important reference for the prediction of therapeutic response and disease progression during NAs therapy (15–22).

In this study, we analyzed the clinical data of 487 patients with CHB and systematically investigated the differences in HBV RNA positivity rates and levels across various treatment statuses, HBsAg levels, HBeAg statuses, and HBV DNA levels. We also explored the correlation between these factors and liver inflammation markers. The goal was to assess the clinical significance of the HBV RNA test in the diagnostic and treatment processes of CHB patients. This research not only enhances the understanding of the pathogenesis of CHB but also provides an important theoretical foundation and practical guidance for optimizing clinical treatment plans, thereby improving treatment outcomes. The findings offer valuable clinical and scientific insights that are crucial for refining treatment strategies and increasing the success rates of CHB therapy.

## 2 Research subjects and methods

### 2.1 Research subjects

This study was conducted on 487 patients with CHB admitted to Jining Public Health Medical Center, and the project was approved by the Medical Ethics Committee of Jining Public Health Medical Center.

### 2.2 Case definition

Inclusion criteria: The enrolled patients were diagnosed according to the criteria outlined in the 2022 Guidelines for the Prevention and Control of Chronic Hepatitis B issued by the Hepatology Branch of the Chinese Medical Association (1). These patients exhibited HBsAg positivity for more than six months, persistent or recurrent ALT abnormalities, and/or significant inflammatory necrosis and/or fibrosis observed in liver histology. Patients in the untreated group had never received antiviral therapy, while patients in the treated group had been on antiviral treatment for more than six months and were currently undergoing treatment.

Exclusion criteria (1): Patients with alcoholic or non-alcoholic fatty liver disease, drug-induced liver disease, autoimmune liver disease, metabolic liver disease, cirrhosis, hepatocellular carcinoma, or infections with hepatotropic viruses other than hepatitis B (e.g., hepatitis A, C, D, or E). (2) Patients with tumors of other organs. (3) Patients with serious cardiovascular, cerebrovascular, pulmonary, renal, endocrine, neurological, or hematologic diseases. (4) Pregnant or breastfeeding women. (5) Patients who are unable to cooperate with treatment due to mental disorders or other reasons.

### 2.3 Methods

#### 2.3.1 Sample collection

Venous blood was collected from each patient in the early morning after an overnight fast. The blood was divided into four 4 mL coagulation-promoting vacuum blood collection tubes, which were left at room temperature until the blood had fully coagulated. The samples were then centrifuged at 2220g for 10 minutes. The resulting serum samples were used for quantitative testing of HBV DNA, HBV RNA, liver function markers, and the five Hepatitis B tests. All samples were collected from the patients simultaneously, and each specimen was handled under strict quality control measures to ensure the accuracy of the tests. To maintain patient confidentiality, all samples were assigned anonymous identification numbers.

#### 2.3.2 Laboratory testing

Serological and biochemical markers: Serum levels of HBsAg and HBeAg were quantified using chemiluminescent immunoassays on an Abbott i2000SR analyzer. Alanine aminotransferase (ALT) and other liver function indicators were measured on a Beckman AU5800 automatic biochemistry analyzer.

HBV DNA quantification: Serum HBV DNA load was quantitatively detected using a commercial real-time PCR kit on a system comprising the Natch S14C automatic nucleic acid extraction system and a real-time fluorescence PCR amplifier, following the manufacturer’s standard protocols.

HBV RNA quantification: Serum HBV RNA was quantified using the commercial Hepatitis B Virus Nucleic Acid Detection Kit (RNA Capture Probe Method). This assay is based on pgRNA sequence-specific RNA capture followed by real-time simultaneous amplification and testing (SAT), an isothermal amplification technology. It specifically targets a conserved region within the HBV RNA sequence, enabling detection across major HBV genotypes (A-H) without cross-reactivity with related viruses (e.g., HCV, HDV). The reaction employs a proprietary enzyme mix containing M-MLV reverse transcriptase and T7 RNA polymerase. After RNA capture and washing,the isothermal amplification was performed at a constant temperature of 42°C for 40 minutes with real-time fluorescence monitoring. Each sample reaction included an internal control (IC); results were considered valid only when the IC signal was positive (IC dt ≤ 30.6, as per kit specifications). Each run incorporated external controls (negative, low-positive, and high-positive quantitative controls), and only runs where all controls met the manufacturer’s validity criteria were accepted. All testing was performed on the dedicated AutoSAT fully automated nucleic acid analysis system to ensure standardization and reproducibility.

### 2.4 Statistical analysis

All the patient information of this this study was collected and summarized in EXCEL through the hospital LIS system, and the data were analyzed and graphed with the help of SPSS 24.0 and GraphPad Prism 9.0 system. Logarithmic transformation was performed for HBV RNA, HBV DNA, HBsAg, and HBeAg levels. Measurement data were tested for normality and expressed as mean ± standard deviation; independent samples t-test was used for comparison between two groups, and one-way analysis of variance (ANOVA) was used for comparison between multiple groups; non-normally distributed data were expressed as *P*50 (*P*25, *P*75), and Mann-Whitney U-test was used for comparison between two groups, and Kruskal-Wallis test was used for comparison between multiple groups. Count data were expressed as frequency (n) and constitutive ratio (%), and the *χ²* test was used for comparisons between groups. For the correlation analysis between two variables, Pearson correlation analysis was used under the premise of satisfying normal distribution and linear relationship, otherwise Spearman correlation analysis was used. The test level *α* = 0.05, and the difference was considered statistically significant at *P* < 0.05.

## 3 Results

### 3.1 Clinical CHB patients with higher HBV RNA positivity than HBeAg and HBV DNA

#### 3.1.1 Positive Rates of HBV RNA, HBeAg, and HBV DNA in 487 CHB patients and in treated and untreated CHB groups

**Figure 1:**
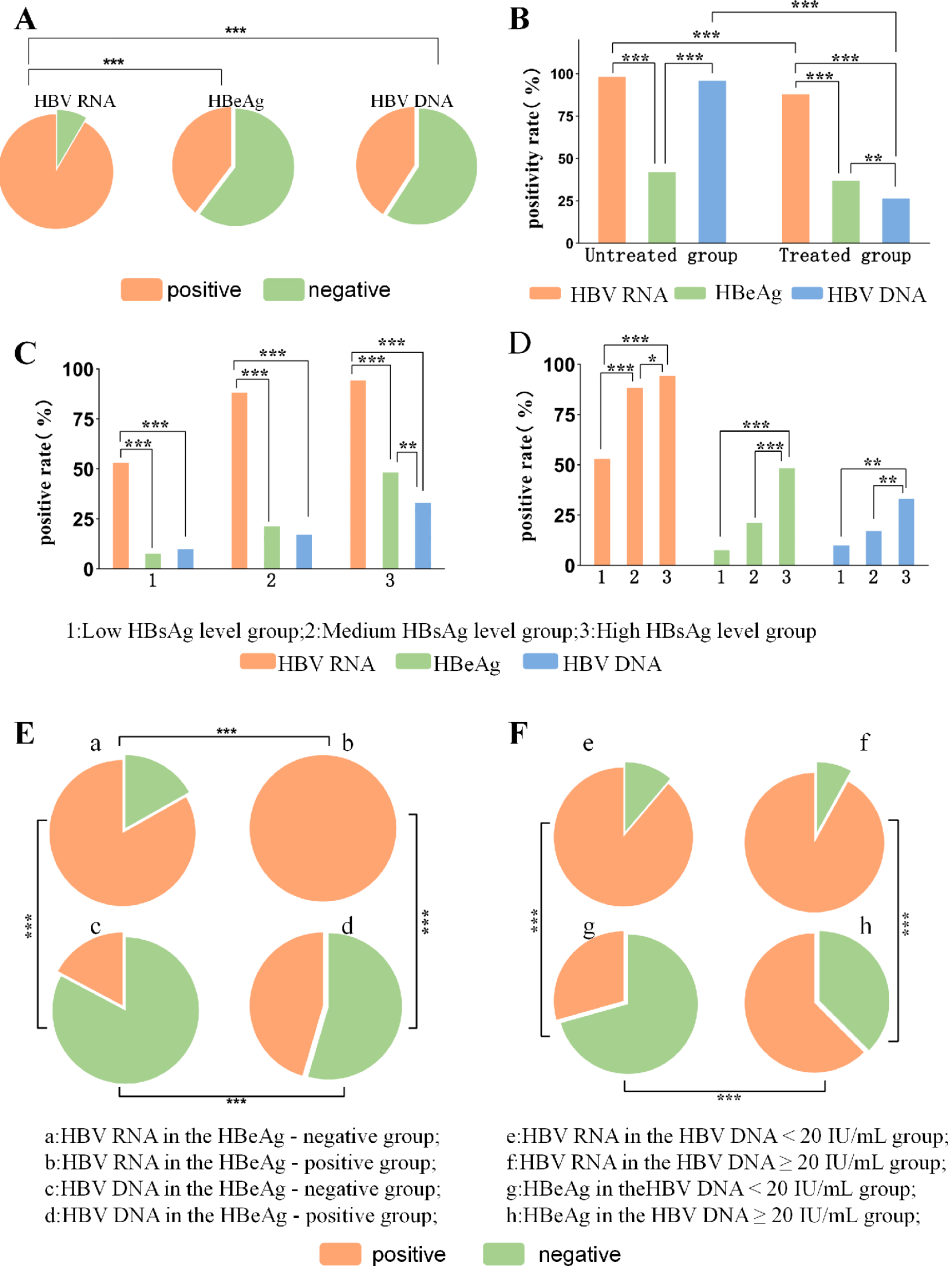
Differential analysis of positivity of HBV RNA, HBeAg and HBV DNA. **(A)** Differential analysis of the positivity rates of HBV RNA, HBeAg, and HBV DNA in 487 patients; **(B)** Differential analysis of the positivity rates of HBV RNA, HBeAg, and HBV DNA in the untreated and treated groups; **(C)** Differential analysis of the positivity rates of HBV RNA, HBeAg, and HBV DNA among the HBsAg level subgroups; **(D)** Differential analysis of the positivity rates of HBV RNA, HBeAg, and HBV DNA among the various HBsAg level subgroups; **(E)** Differential analysis of the positivity rates of HBV RNA and HBV DNA in the HBeAg-negative and HBeAg-positive groups; and **(F)**. HBV RNA in the HBV DNA < 20 IU/mL group and HBV DNA ≥ 20 IU/mL group, differential analysis of the positivity rate of HBeAg. *P < 0.05; **P < 0.01; ***P < 0.001.

The *χ²* test revealed that among the 487 CHB patients included, the positivity rate of HBV RNA was significantly higher than the positivity rate of HBeAg and HBV DNA, and there was no significant difference in the positivity rate of HBeAg and HBV DNA (**Figure 1A**). These patients were divided into the treated but not yet cured group and the untreated group. In the untreated group, the positivity rates of HBV DNA and HBV RNA were significantly higher than the positivity rates of HBeAg, and there was no significant difference between the two rates of HBV DNA and HBV RNA, which indicated that in untreated patients, HBV DNA and HBV RNA both reflected the state of viruses and both had similar detection value (22). In the treated group, the positive rate of HBV RNA was significantly higher than that of HBV DNA and HBeAg, and the positive rate of HBeAg was higher than that of HBV DNA, indicating that the viral transcription may be suppressed after antiviral therapy, and that the transcriptional activity of cccDNA persisted when the HBV DNA test was lower than the lower limit of the HBeAg test or when HBeAg was negative, which suggests that the HBV RNA of the treated group can more sensitively reflect the state of viruses. It suggests that HBV RNA in the treated group is more sensitive to the state of viruses. The positivity rates of HBV RNA and HBV DNA in the treated group were significantly lower than those in the untreated group, suggesting that the treatment had a significant impact on viral replication and transcriptional activity, and that the viral replication level of the treated patients was generally reduced (**Figure 1B**). Next, patients in the treated group were grouped according to HBsAg, HBeAg and HBV DNA status, and the differences in the positivity rates of HBV RNA and other indexes of treated patients in each group were further analyzed.

#### 3.1.2 Patients with CHB in the treated group, categorized based on HBsAg, HBeAg, and HBV DNA levels, all exhibited a high rate of HBV RNA positivity

Of the 398 treated CHB patients, 257 were in the high HBsAg level (> 1000 IU/mL) group, 97 in the intermediate HBsAg level (100 ~ 1000 IU/mL) group, and 44 in the low HBsAg level (< 100 IU/mL) group. The HBV RNA positivity rate was significantly higher than that of HBV DNA and HBeAg in CHB patients grouped by high, intermediate, and low HBsAg levels (**Figure 1C**), suggesting that HBV RNA reflects HBV transcriptional activity more sensitively than HBV DNA and HBeAg at all levels of HBsAg. The highest positivity rate of HBV RNA was observed in the group with high HBsAg levels, followed by the medium HBsAg group, and the lowest in the low HBsAg group. The positivity rates of HBeAg and HBV DNA were significantly higher in the high HBsAg level group than in the intermediate and low HBsAg level groups, but there was no significant difference between the positivity rates in the intermediate HBsAg level group and the low HBsAg level group, indicating that HBeAg, HBV DNA were not sufficiently susceptible to the low level of viral activity (**Figure 1D**). Possible reasons for this are hypothesized to be: a: Despite treatment, a large proportion of the population remains in a high HBsAg state with active viral transcription, requiring intensive antiviral therapy to suppress cccDNA activity. b: Even at low HBsAg levels, HBV may persistently replicate at low levels. HBV RNA can help identify this potentially replicating population, which may be misclassified as “low risk” based on traditional markers such as HBeAg or HBV DNA. These patients are, in fact, still at risk of disease progression or transmission and should be carefully evaluated before discontinuing treatment. In order to further analyze this group of patients misclassified as “low risk” by HBeAg or HBV DNA, we further grouped HBeAg and HBV DNA levels in the treated group, and further explored the unique clinical value of HBV RNA.

Of the 398 treated CHB patients, 244 were in the HBeAg-negative group, while 154 were in the HBeAg-positive group. In both groups, the rate of HBV RNA positivity was significantly higher than that of HBV DNA positivity. The positive rates of both HBV RNA and HBV DNA were significantly higher in the HBeAg-positive group compared to the HBeAg-negative group (**Figure 1E**). This finding highlights the high sensitivity and clinical value of HBV RNA in monitoring viruses. It also suggests that the level of viral activity and the risk of disease progression vary according to the patient’s HBeAg status.

Among the 398 treated CHB patients, 286 were in the HBV DNA < 20 IU/mL group, and 112 were in the HBV DNA ≥ 20 IU/mL group. In both groups, the rate of HBV RNA positivity was significantly higher than the rate of HBeAg positivity. Moreover, no significant difference was observed in the HBV RNA positivity rate between the two groups (**Figure 1F**). This suggests that during antiviral therapy, HBV DNA levels may decrease due to drug inhibition. Relying solely on HBV DNA monitoring in such cases may underestimate the risk of viral persistence. HBV RNA, however, can still detect low levels of viral transcription, emphasizing its role as a sensitive marker for viruses. This makes HBV RNA a valuable tool to fill the “blind spot” left by HBV DNA monitoring.

In conclusion, the high sensitivity and stability of HBV RNA make it a valuable diagnostic and monitoring tool across various clinical scenarios—such as in untreated patients, treated patients with intermediate HBsAg levels, low HBsAg levels, HBeAg negativity, and low HBV DNA levels. HBV RNA can serve as an important complement to traditional markers like HBV DNA and HBeAg, providing a more comprehensive assessment of a patient’s condition and treatment response, and offering a foundation for developing individualized treatment plans.

### 3.2 Detection levels of HBV RNA, HBeAg and HBV DNA

#### 3.2.1 Analysis of the detection level of each index in 487 CHB patients and CHB patients in the treated and untreated groups

Based on the data of 487 patients and these patients in the untreated and treated groups, it canbe seen that the serum HBV RNA, HBV DNA, HBeAg, HBsAg levels, as well as ALT and AST of the patients in the treated group were significantly lower than those in the untreated group (**Supplementary Figure S1**), which indicates that antiviral therapy can effectively inhibit viral replication and improve hepatic inflammation. In patients in the treated group, the HBV RNA level was 2.72 log10 copies/mL in a state where HBV DNA and HBeAg levels were 0 (**Table 1**), which indicated that low levels of viral transcription still existed *in vivo* even when HBV DNA and HBeAg were undetectable, suggesting that continuous monitoring of HBV RNA levels was still needed in these patients in order to detect potential viral activity and reduce the risk of liver disease progression. In the next step, patients in the treated group will be grouped according to HBsAg, HBeAg and HBV DNA status, and the differences in the levels of HBV RNA and other indicators detected in the treated patients in each group will be further analyzed.

**Table 1:** General information of 487 patients and of patients in the untreated and treated groups.

Item	Enrolled patients	Untreated group	Treated group	*P* value
Patient distribution	487(100%)	89(18.3%)	389(71.7%)	–
Proportion of males	344(70.6%)	56(62.9%)	288(72.4%)	0.094
Age (years)	41 (35,48)	39.00(34.00, 49.50)	41(36.00,48.00)	0.329
ALT(U/L)	27.50(17.60,46.40)	43.60(24.30, 103.15)	26.40(17.00,39.23)	**<0.001**
AST(U/L)	26.10(19.80,36.90)	38.10(23.95, 63.40)	24.90(19.40,32.80)	**<0.001**
GGT(U/L)	26.90(17.60,50.60)	25.90(16.10, 56.95)	27.00(18.00,48.43)	0.878
TBIL(μmol/L)	14.00(11.00,17.90)	15.10(11.05, 20.35)	13.85(10.88,17.43)	0.173
ALB(g/L)	45.01 ± 3.14	44.45 ± 3.84	45.14 ± 2.95	0.115
HBV RNA(log_10_ copies/mL)	2.93(2.04, 4.76)	4.90(2.09, 7.54)	2.72(2.04,4.18)	**<0.001**
HBsAg(log_10_ IU/mL)	3.34(2.86, 3.91)	3.77(3.26, 4.32)	3.26(2.77,3.78)	**0.001**
HBeAg(log_10_ PEIU/mL)	0(0.00,0.61)	0(0, 2.81)	0(0, 0.44)	**<0.001**
HBV DNA(log_10_ IU/mL)	0(0.00,2.17)	5.12(3.60, 8.21)	0(0,1.39)	**<0.001**

The chi-square test was used for between-group comparisons of the proportion of males; the Mann-Whitney U test was used for between-group comparisons of age, ALT, AST, TBIL, HBV RNA, HBsAg, HBeAg, and HBV DNA levels, and the t-test was used for between-group comparisons of ALB. Bold numbers indicate p < 0.05 (statistically significant).

#### 3.2.2 Analysis of the level of detection of each indicator in the treated group of CHB patients in the HBsAg, HBeAg and HBV DNA level subgroups of CHB patients

As shown in **Table 2** and **Supplementary Figure S2**, among patients receiving treatment, HBV RNA showed a positive correlation with HBsAg grouping, while HBeAg and HBV DNA were difficult to detect. This suggests that HBV RNA can still reflect the transcriptional activity of cccDNA after treatment, thereby compensating for the limitations of HBeAg and HBV DNA in assessing low-level viral replication.

**Table 2:** General information of patients grouped by HBsAg level in the treated group of patients.

Item	Low HBsAg level group	Medium HBsAg level group	High HBsAg level group	*P* value
Patient distribution	44(11.1%)	97(24.4%)	257(64.5%)	–
Proportion of males	35(79.5%)	68(70.1%)	185(72.0%)	0.496
Age (years)	42.00(37.50, 54.75)	42.00(35.00,52.00)	41.00(36.00,46.00)	0.120
ALT(U/L)	27.80(19.25, 53.05)	24.80(15.30,41.50)	26.70(17.25,38.10)	0.542
AST(U/L)	26.15(19.53, 38.50)	25.50(19.55,36.10)	24.40(19.30,31.50)	0.287
GGT(U/L)	37.20(20.03, 54.95)	26.50(17.40,55.90)	26.90(17.75,44.75)	0.284
TBIL(μmol/L)	14.95(12.23, 19.23)	14.20(11.00,18.30)	13.50(10.65,16.95)	0.083
ALB(g/L)	45.08 ± 3.26	44.70 ± 2.78	45.32 ± 2.95	0.182
HBV RNA(log_10_ copies/mL)	2(0, 2.40)	2.59(2.01,3.50)	3.24(2.22,4.94)	**<0.001**
HBsAg(log_10_ IU/mL)	1.43(0.82, 1.80)	2.68(2.30,2.90)	3.62(3.28,3.99)	**<0.001**
HBeAg(log_10_ PEIU/mL)	0 (0, 0)	0(0, 0)	0(0, 1.11)	**<0.001**
HBV DNA(log_10_ IU/mL)	0(0, 0)	0(0, 0)	0(0,1.63)	**<0.001**

The chi-square test was used for between-group comparisons of the proportion of males, HBV RNA, HBeAg, and HBV DNA positivity rates; the Kruskal-Wallis test was used for between-group comparisons of age, ALT, AST, TBIL, HBV RNA, HBsAg, HBeAg, and HBV DNA levels, and a one-way analysis of variance (ANOVA) was used for between-group comparisons of ALB. Bold numbers indicate p < 0.05 (statistically significant).

Patients in the treated group were grouped by HBeAg level, and the serum ALT, TBIL, HBV RNA, HBVDNA, and HBsAg levels were higher in the HBeAg-positive group than in the HBeAg-negative group(**Supplementary Figure S3**). In the HBeAg-negative group, the HBV RNA level was 2.17 log10 copies/mL in the state where the HBV DNA detection level was 0 log10 IU/mL (**Table 3**), indicating that even if the HBV DNA was undetectable, the transcriptional activity of the HBV virus still existed, further confirming that the HBV RNA could make up for the HBeAg, HBV DNA insufficiency and more sensitively respond to the state of the virus.

**Table 3:** General information of patients grouped by HBeAg level in the treated group of patients.

Item	HBeAg negative group	HBeAg positive group	*P* value
Patient distribution	244(61.3%)	154(38.7%)	–
Proportion of males	171(70.1%)	117(76.0%)	0.200
Age (years)	41(35,49)	41(37,47)	0.941
ALT(U/L)	24.25(16.13,37.20)	29.60(18.15,42.35)	**0.019**
AST(U/L)	24.10(19.40,31.40)	25.55(19.70,34.88)	0.22
GGT(U/L)	26.30(17.53,45.75)	30.45(18.28,59.08)	0.157
TBIL(μmol/L)	13.30(10.60,17.00)	14.75(11.38,18.50)	**<0.001**
ALB(g/L)	45.08 ± 2.97	45.23 ± 2.92	0.619
HBV RNA(log_10_ copies/mL)	2.17(2.00,2.71)	4.56(3.65,6.39)	**<0.001**
HBsAg(log_10_ IU/mL)	3.06(2.29,3.50)	3.64(3.17,4.09)	**<0.001**
HBeAg(log_10_ PEIU/mL)	–	0.76(0.23,2.08)	**-**
HBV DNA(log_10_ IU/mL)	0(0,0)	0(0,1.80)	**<0.001**

The chi-square test was used for between-group comparisons of the proportion of males, HBV RNA, HBeAg, and HBV DNA positivity rates; the Mann-Whitney U test was used for between-group comparisons of age, ALT, AST, TBIL, HBV RNA, HBsAg, HBeAg, and HBV DNA levels, and the t-test was used for between-group comparisons of ALB. Bold numbers indicate p < 0.05 (statistically significant).

Grouped by HBV DNA levels, serum ALT, AST, GGT, HBV RNA, HBsAg, and HBeAg were significantlyhigher in the HBV DNA ≥ 20 IU/mL group than in the HBV DNA < 20 IU/mL group (**Supplementary Figure S4**). While in the HBV DNA < 20 IU/mL group, HBeAg was detected as 0 log10 PEIU/mL in the state of HBV RNA level was 2.47 log10 copies/mL (**Table 4**), indicating that antiviral therapy suppressed viral replication to some extent, but the persistent positivity of HBV RNA suggested that the transcriptional activity of the virus was not completely suppressed.

**Table 4:** General information of patients grouped by HBV DNA level in the treated group of patients .

Item	HBV DNA < 20IU/mL group	HBV DNA ≥ 20IU/mL group	*P* value
Patient distribution	286(71.9%)	112(28.1%)	–
Proportion of males	206(72.0%)	82(73.2%)	0.812
Age (years)	42(36,49)	40(35.00,45.00)	0.057
ALT(U/L)	24.10(15.45,35.65)	33.05(22.00,55.80)	**<0.001**
AST(U/L)	23.70(18.83,30.88)	27.80(21.85,45.43)	**<0.001**
GGT(U/L)	24.70(17.28,45.20)	33.80(20.43,65.78)	**0.001**
TBIL(μmol/L)	13.85(11.10,17.53)	13.60(10.63,17.25)	0.45
ALB(g/L)	45.10(43.40,46.93)	45.25(43.03,47.10)	0.591
HBV RNA(log_10_ copies/mL)	2.47(2.00,3.60)	4.24(2.41,6.61)	**<0.001**
HBsAg(log_10_ IU/mL)	3.16(2.53,3.63)	3.72(3.05,4.26)	**<0.001**
HBeAg(log_10_ PEIU/mL)	0(0,0.003)	0.47(0,2.34)	**<0.001**
HBV DNA(log_10_ IU/mL)		1.91(1.49,2.65)	**<0.001**

The chi-square test was used for between-group comparisons of the proportion of males, HBV RNA, HBeAg, and HBV DNA positivity rates; the Mann-Whitney U test was used for between-group comparisons of age, ALT, AST, TBIL, ALB, HBV RNA, HBsAg, HBeAg, and HBV DNA levels. Bold numbers indicate p < 0.05 (statistically significant).

In summary, the performance of HBV RNA levels in both treated and untreated groups, as well as across various subgroups categorized by HBsAg, HBeAg, and HBV DNA status, suggests that HBV RNA can complement HBV DNA and HBeAg. It serves as a crucial indicator of viral transcriptional activity. In clinical practice, monitoring HBV RNA levels should be prioritized to guide the development of individualized treatment strategies.

### 3.3 Correlation analysis of HBV RNA, HBeAg and HBV DNA

After differentially analyzing the HBV RNA levels of the patients in each group, we found that they showed significant differences in different subgroups. To further explore the potential associations between these differences and indicators such as the degree of active viral replication and the severity of hepatic inflammatory injury, we next performed a correlation analysis. Through Spearman’s correlation analysis we found positive correlations between ALT, AST, and GGT in all subgroups, suggesting that these virologic markers are consistent in reflecting the severity of hepatic inflammation and injury and are less susceptible to the effects of treatment and viral load. Positive correlations between serum HBV RNA, HBsAg, HBeAg, and HBV DNA were found in the untreated patient group as well as in the high HBsAg level group, HBeAg-positive group, and HBV DNA ≥ 20 IU/mL group. In the treated patient group, positive correlations were also found, including in the intermediate and low HBsAg level groups, HBeAg-negative group, and HBV DNA < 20 IU/mL group. These findings suggest that the correlation between HBV RNA, HBsAg, HBeAg, and HBV DNA reliably reflects the state of viral replication. However, this correlation is influenced by antiviral therapy.

**Figure 2:**
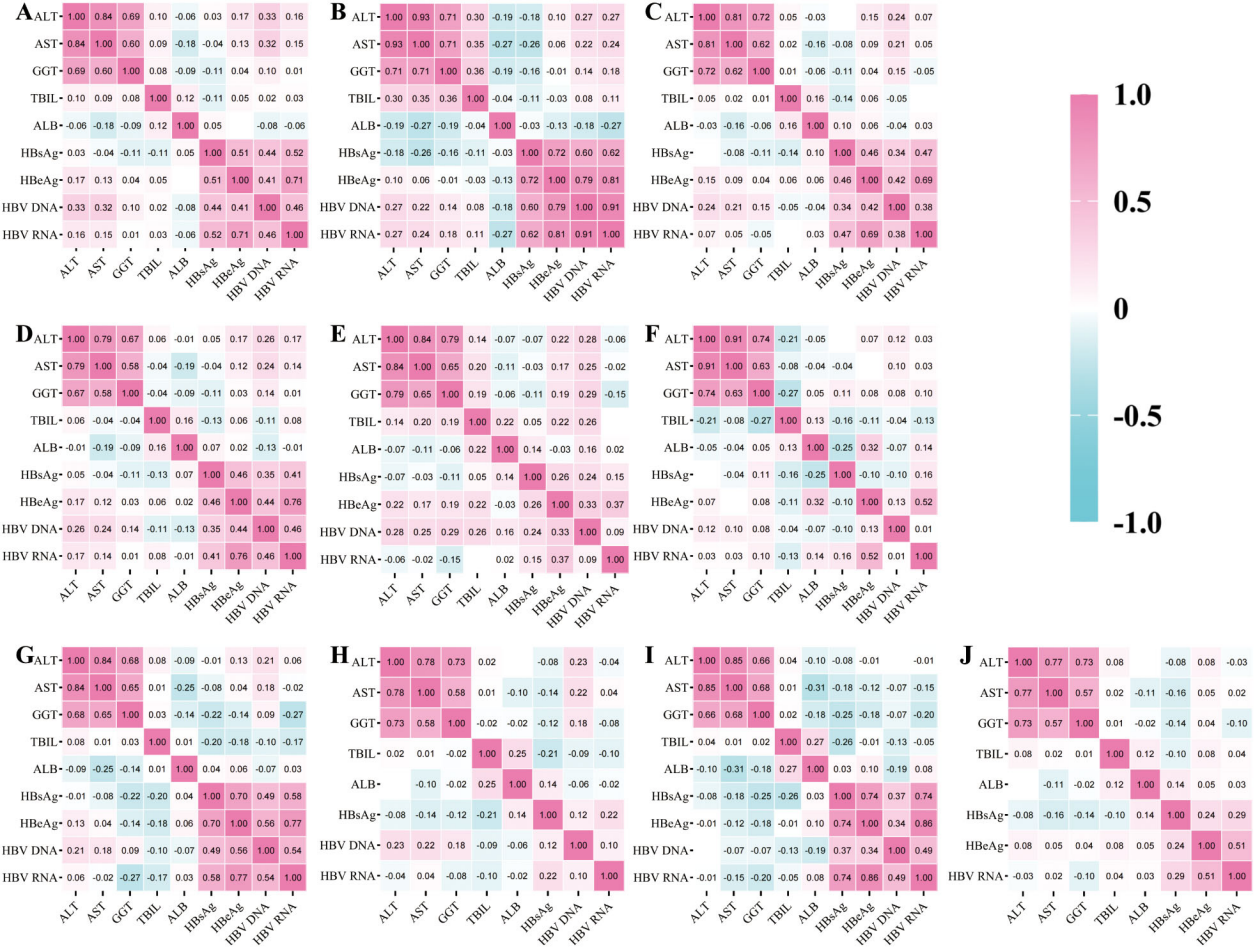
Heatmap of correlation analysis between HBV RNA and other related indicators. **(A)** Correlation analysis among indicators of 487 CHB patients; **(B)** Correlation analysis among indicators of CHB patients in the untreated group; **(C)** Correlation analysis among indicators of patients in the treated group; **(D)** Correlation analysis among indicators of patients in the group with high HBsAg level; **(E)** Correlation analysis among indicators of patients in the group with moderate HBsAg level; **(F)** Correlation analysis among indicators of patients in the group with low HBsAg level group patients’ correlation analysis of each indicator; **(G)** correlation analysis of each indicator in the HBeAg positive group patients; **(H)** correlation analysis of each indicator in the HBeAg negative group patients; **(I)** correlation analysis of each indicator in the HBV DNA ≥ 20IU/mL group patients; **(J)** correlation analysis of each indicator in the HBV DNA < 20IU/mL group patients).

In the untreated group of patients as well as the high HBsAg level group, HBeAg positive group, HBV DNA ≥ 20 IU/mL group of patients serum HBV RNA and ALT, AST, HBsAg, HBeAg, HBV DNA have correlation, indicating that when viruses are active, the degree of hepatic inflammation and injury is more serious suggesting that HBV RNA can reflect both hepatic inflammation and viral transcription status; meanwhile, the correlation between serum HBV RNA and ALT, AST, HBsAg, HBeAg, HBV DNA was weakened in the treated patients as well as in the group with moderate HBsAg level, low HBsAg level, HBeAg-negative group, and the group with HBV DNA < 20 IU/mL, suggesting that HBV RNA has a unique detection value in the patients with post-treatment and low viral load (**Figure 2**).

## 4 Discussion

CHB represents a significant global public health challenge and poses a serious threat to human health (2). The primary goals of CHB therapy are to suppress viral replication, reduce hepatic inflammation, and lower the risk of cirrhosis and HCC (1). The clinical efficacy of NAs as the drug of choice for CHB treatment has been well established in several studies. Studies have shown that NAs significantly inhibit HBV replication by inhibiting viral DNA polymerase activity, alleviate hepatic inflammatory response, and improve liver histopathological changes in cirrhotic patients (23–26). However, the mechanism of action of NAs determines their inability to act directly on cccDNA, resulting in the persistence and maintenance of low levels of transcriptional activity of cccDNA in hepatocytes, which poses a great challenge for the complete cure of CHB (27). Therefore, in the management of CHB, accurate assessment of viral replication is essential for developing effective therapeutic strategies and making prognostic judgments. Since cccDNA is primarily located in the nucleus of hepatocytes and there is a lack of standardized, widely applicable clinical assays, there is an urgent need to identify alternative markers that can accurately reflect the transcriptional activity of cccDNA. In this paper, we explored the potential value of HBV RNA in individualized treatment, efficacy monitoring, and prognosis determination in CHB by examining its performance across different patient subgroups.

We observe that HBV RNA maintains a high detection rate and level in patients with HBV DNA below the detection limit or in those who are HBeAg-negative. This suggests that HBV RNA more accurately reflects the transcriptional activity of cccDNA in hepatocytes within the patient. This important finding aligns closely with the results of Lai et al (28), who, in their study of 43 patients with subdetectable levels of HBV DNA after antiviral therapy, identified 22 patients with negative intrahepatic cccDNA, 17 patients with negative serum HBV RNA, and 12 patients with both negative cccDNA and HBV RNA. Wang et al. (22) also found that HBV RNA could still be detected in 35 out of 47 CHB patients, even when the level of HBV DNA was below the lower limit of detection after treatment. This suggests that serum HBV RNA offers distinct advantages in monitoring the status of viruses. The conventional view holds that HBV DNA negativity is indicative of effective antiviral therapy. However, the results of this study suggest that relying solely on HBV DNA testing may underestimate the true state of viruses. Moreover, HBV RNA holds significant value in predicting HBV viral relapse and disease progression. Wang et al. (29) followed up CHB patients who had met termination criteria after NAs treatment after discontinuation. The results showed that 21 out of 44 patients who were seropositive for HBV RNA at the time of discontinuation experienced viral relapse after discontinuation, whereas only 3 out of 12 patients who were negative for HBV RNA at the time of discontinuation experienced relapse after discontinuation. Xia et al. (16) also found that patients with HBV RNA levels ≥ 20,000 copies/mL at the end of treatment experienced biochemical relapses up to 6 years after discontinuation of the drug, and that the risk of relapse was significantly lower in patients with lower HBV RNA levels. The present study further highlights the potential of HBV RNA as a valuable biomarker for predicting relapse after drug discontinuation, based on a larger sample size of clinical data. By measuring serum HBV RNA levels at the end of treatment, it is possible to predict the risk of relapse following drug cessation, thereby providing clinicians with a useful reference for making decisions about drug discontinuation.

The results of this study demonstrated that, after NAs treatment, patients experienced a significant decrease in serum HBV RNA, HBV DNA, HBsAg, HBeAg levels, and liver function markers (ALT, AST). This suggests that NAs treatment effectively inhibited viral replication, thereby reducing liver inflammation. The continuous replication of HBV can stimulate the immune response, leading to hepatocellular damage and subsequent liver function abnormalities. Therefore, the normalization of liver function markers following treatment serves as a crucial indicator of the effectiveness of antiviral therapy. This finding is consistent with the findings of Marcellin et al. (30), whose study showed that entecavir (ETV) treatment significantly reduced HBV DNA levels and improved liver function indices (ALT, AST, etc.). Meanwhile the normalization of liver function was closely related to long-term prognosis. A study by Liaw et al. (31) confirmed that the risk of cirrhosis and hepatocellular carcinoma was significantly lower in patients with sustained viral suppression and normal liver function. However, this study found that the serum HBV RNA test showed a high positivity rate, with HBV still exhibiting replicative activity, even when liver function remained within the normal range. This further confirms that HBV RNA can serve as a more sensitive indicator for assessing the state of viruses. Further correlation analysis revealed a significant relationship between serum HBV RNA levels and markers such as ALT, AST, HBsAg, HBeAg, and HBV DNA. This suggests that HBV RNA not only reflects the degree of viral transcription but also the status of the patient’s liver function. Additionally, this study found significant differences in HBV RNA levels and related markers across different treatment stages and virological states. These findings highlight the dynamic changes in HBV RNA, which can effectively distinguish patients with varying virological statuses and reflect both viral transcription activity and liver damage in patients with different viral loads.

In conclusion, the high positivity rate of serum HBV RNA indicates that its detection holds significant clinical value in the overall management of patients with CHB.

## 4 Limitations and future research directions

### 4.1 Theoretical limitation regarding the origin and significance of serum HBV RNA and future direction

The detected serum HBV RNA may be influenced by residual transcripts produced during the early stages of infection and may not fully reflect the presence of intrahepatic cccDNA. To clarify its compositional origins and its real-time correlation with the transcriptional activity of intrahepatic cccDNA, further studies will incorporate data from intrahepatic tissue biopsy cccDNA assays to more accurately elucidate the clinical significance of serum HBV RNA.

### 4.2 Limitation in generalizability due to the single-center cohort and future direction

The study sample was drawn from a single center, which, while ensuring consistency in data quality control, may limit the generalizability of the findings. To enhance the representativeness and reliability of the research, we plan to conduct multicenter collaborative studies involving broader and more diverse populations to validate and extend the preliminary findings of this study.

## Data Availability

The raw data supporting the conclusions of this article will be made available by the authors, without undue reservation.

## References

[1] Chinese Society of Hepatology Chinese Medical Association Chinese Society of Infectious Diseases Chinese Medical Association . Guidelines for the prevention and treatment of chronic hepatitis B (version 2022). Chin J Hepatol. (2022) 30:1309–31. doi: 10.3760/cma.j.cn501113-20221204-00607, PMID: 36891718 PMC12677433

[2] World Health Organization . Global progress report on HIV, viral hepatitis and sexually transmitted infections, 2021: accountability for the global health sector strategies 2016–2021: actions for impact. 1st ed. Geneva: World Health Organization (2021).

[3] Wang Y . Establishing a public health management model to promote the elimination of viral hepatitis threats. J Clin Hepatol. (2025) 41:201–4. doi: 10.12449/JCH250201

[4] Hong X Kim ES Guo H . Epigenetic regulation of hepatitis B virus covalently closed circular DNA: implications for epigenetic therapy against chronic hepatitis B. Hepatology. (2017) 66:2066–77. doi: 10.1002/hep.29479, PMID: 28833361 PMC5696023

[5] Marchetti AL Guo H . New insights on molecular mechanism of hepatitis B virus covalently closed circular DNA formation. Cells. (2020) 9:2430. doi: 10.3390/cells9112430, PMID: 33172220 PMC7694973

[6] Erken R Loukachov V van Dort K van den Hurk A Takkenberg RB de Niet A . Quantified integrated hepatitis B virus is related to viral activity in patients with chronic hepatitis B. Hepatology. (2022) 76:196–206. doi: 10.1002/hep.32352, PMID: 35073596 PMC9305117

[7] Zoulim F Chen PJ Dandri M Kennedy PT Seeger C . Hepatitis B virus DNA integration: implications for diagnostics, therapy, and outcome. J Hepatol. (2024) 81:1087–99. doi: 10.1016/j.jhep.2024.06.037, PMID: 38971531

[8] Liang X Xie Q Shang J Tang H Xu M Meng QH . Tenofovir disoproxil fumarate for multiple nucleos(t)ide analogues treatment failure hepatitis B: is monotherapy enough? J Gastroenterol Hepatol. (2022) 37:471–9. doi: 10.1111/jgh.15757, PMID: 34894002 PMC9303406

[9] Lee HW Park JY Lee JW Yoon KT Kim CW Park H . Long-term efficacy of tenofovir disoproxil fumarate monotherapy for multidrug-resistant chronic HBV infection. Clin Gastroenterol Hepatol. (2019) 17:1348–1355.e2. doi: 10.1016/j.cgh.2018.10.037, PMID: 30613003

[10] Liu Y Corsa AC Buti M Cathcart AL Flaherty JF Miller MD . No detectable resistance to tenofovir disoproxil fumarate in HBeAg+ and HBeAg– patients with chronic hepatitis B after 8 years of treatment. J Viral Hepat. (2017) 24:68–74. doi: 10.1111/jvh.12613, PMID: 27658343

[11] Chen CH Peng CY Kuo YH Hu TH Hung CH Wang JH . Earlier and higher rate of hepatitis B virus relapse after discontinuing tenofovir versus entecavir in hepatitis B e antigen–positive patients. J Infect Dis. (2022) 225:1974–81. doi: 10.1093/infdis/jiab596, PMID: 34894128

[12] Liu YC Jeng WJ Peng CW Chien RN Liaw YF . Off-tenofovir hepatitis flares in HBeAg-negative patients occur earlier, more frequent and severe than those off-entecavir therapies. Liver Int. (2022) 42:551–60. doi: 10.1111/liv.15140, PMID: 34936719

[13] Nassal M . HBV cccDNA: viral persistence reservoir and key obstacle for a cure of chronic hepatitis B. Gut. (2015) 64:1972–84. doi: 10.1136/gutjnl-2015-309809, PMID: 26048673

[14] Nguyen MH Wong G Gane E Kao JH Dusheiko G . Hepatitis B virus: advances in prevention, diagnosis, and therapy. Clin Microbiol Rev. (2020) 33:e00046–19. doi: 10.1128/CMR.00046-19, PMID: 32102898 PMC7048015

[15] Cortese MF Riveiro-Barciela M Tabernero D Rodriguez-Algarra F Palom A Sopena S . Standardized hepatitis B virus RNA quantification in untreated and treated chronic patients: a promising marker of infection follow-up. Microbiol Spectr. (2022) 10:e02149–21. doi: 10.1128/spectrum.02149-21, PMID: 35377229 PMC9045303

[16] Xia M Chi H Wu Y Hansen BE Li Z Liu S . Serum hepatitis B virus RNA level is associated with biochemical relapse in patients with chronic hepatitis B infection who discontinue nucleos(t)ide analogue treatment. Aliment Pharmacol Ther. (2021) 54:709–14. doi: 10.1111/apt.16538, PMID: 34275138

[17] Wu Y Wen J Tang G Zhang J Xin J . On-treatment HBV RNA dynamic predicts entecavir-induced HBeAg seroconversion in children with chronic hepatitis B. J Infect. (2021) 83:594–600. doi: 10.1016/j.jinf.2021.08.044, PMID: 34474058

[18] Wang X Chi X Wu R Xu H Gao X Yu L . Serum HBV RNA correlated with intrahepatic cccDNA more strongly than other HBV markers during peg-interferon treatment. Virol J. (2021) 18:4. doi: 10.1186/s12985-020-01471-2, PMID: 33407619 PMC7789711

[19] van Campenhout MJH van Bömmel F Pfefferkorn M Fischer J Deichsel D Boonstra A . Serum hepatitis B virus RNA predicts response to peginterferon treatment in HBeAg-positive chronic hepatitis B. J Viral Hepat. (2020) 27:610–9. doi: 10.1111/jvh.13272, PMID: 32052503 PMC7383601

[20] Luo H Zhang XX Cao LH Tan N Kang Q Xi HL . Serum hepatitis B virus RNA is a predictor of HBeAg seroconversion and virological response with entecavir treatment in chronic hepatitis B patients. World J Gastroenterol. (2019) 25:719–28. doi: 10.3748/wjg.v25.i6.719, PMID: 30783375 PMC6378541

[21] Liu Y Jiang M Xue J Yan H Liang X . Serum HBV RNA quantification: useful for monitoring natural history of chronic hepatitis B infection. BMC Gastroenterol. (2019) 19:53. doi: 10.1186/s12876-019-0966-4, PMID: 30991954 PMC6469196

[22] Ghany MG King WC Lisker-Melman M Lok ASF Terrault N Janssen HLA . Comparison of HBV RNA and hepatitis B core related antigen with conventional HBV markers among untreated adults with chronic hepatitis B in North America. Hepatology. (2021) 74:2395–409. doi: 10.1002/hep.32018, PMID: 34133774 PMC8895675

[23] Lampertico P Agarwal K Berg T Buti M Janssen HLA Papatheodoridis G . EASL 2017 clinical practice guidelines on the management of hepatitis B virus infection. J Hepatol. (2017) 67:370–98. doi: 10.1016/j.jhep.2017.03.021, PMID: 28427875

[24] Terrault NA Lok ASF McMahon BJ Chang KM Hwang JP Jonas MM . Update on prevention, diagnosis, and treatment of chronic hepatitis B: AASLD 2018 hepatitis B guidance. Clin Liver Dis. (2018) 12:33–4. doi: 10.1002/cld.728, PMID: 30988907 PMC6385899

[25] Hou JL Zhao W Lee C Hann HW Peng CY Tanwandee T . Outcomes of long-term treatment of chronic HBV infection with entecavir or other agents from a randomized trial in 24 countries. Clin Gastroenterol Hepatol. (2020) 18:457–467.e21. doi: 10.1016/j.cgh.2019.07.010, PMID: 31306800

[26] Lai CL Chang TT DeHertogh D Hadziyannis SJ Cianciara J Rizzetto M . Entecavir versus lamivudine for patients with HBeAg-negative chronic hepatitis B. N Engl J Med. (2006) 354:1011–20. doi: 10.1056/NEJMoa051287, PMID: 16525138

[27] Revill PA Penicaud C Brechot C Zoulim F . Meeting the challenge of eliminating chronic hepatitis B infection. Genes. (2019) 10:260. doi: 10.3390/genes10040260, PMID: 30939846 PMC6523454

[28] Lai CL Wong D Ip P Kopaniszen M Seto WK Fung J . Reduction of covalently closed circular DNA with long-term nucleos(t)ide analogue treatment in chronic hepatitis B. J Hepatol. (2017) 66:275–81. doi: 10.1016/j.jhep.2016.08.022IF, PMID: 27639844

[29] Wang J Shen T Huang X Kumar GR Chen X Zeng Z . Serum hepatitis B virus RNA is encapsidated pregenome RNA that may be associated with persistence of viral infection and rebound. J Hepatol. (2016) 65:700–10. doi: 10.1016/j.jhep.2016.05.029, PMID: 27245431

[30] Marcellin P Heathcote EJ Buti M Gane E de Man RA Krastev Z . Tenofovir disoproxil fumarate versus adefovir dipivoxil for chronic hepatitis B. N Engl J Med. (2008) 359:2442–55. doi: 10.1056/NEJMoa0802878, PMID: 19052126

[31] Liaw YF Sung JJY Chow WC Farrell G Lee CZ Yuen H . Lamivudine for patients with chronic hepatitis B and advanced liver disease. N Engl J Med. (2004) 351:1521–31. doi: 10.1056/NEJMoa033364, PMID: 15470215

