# Supplementary material for: Clinical superiority of HBV RNA testing in patients with chronic hepatitis B
Source: Front Immunol. 2026 Feb 25;17:1689229. doi: 10.3389/fimmu.2026.1689229 (PMC12975957; doi:10.3389/fimmu.2026.1689229)
Supplement: Supplementary file 1 [file Image1.pdf]

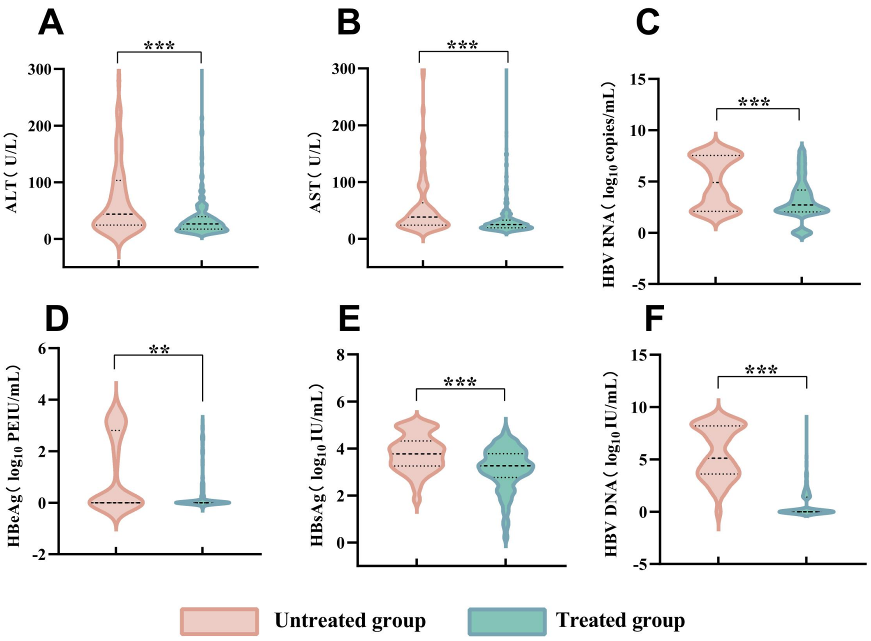

Fig. S1. Indicators with significant differences between the untreated group and the treated group (A: ALT; B: AST; C: HBV RNA; D: HBeAg; E:HBsAg; F: HBV DNA). **, *P* < 0.01; ***, *P* < 0.001.

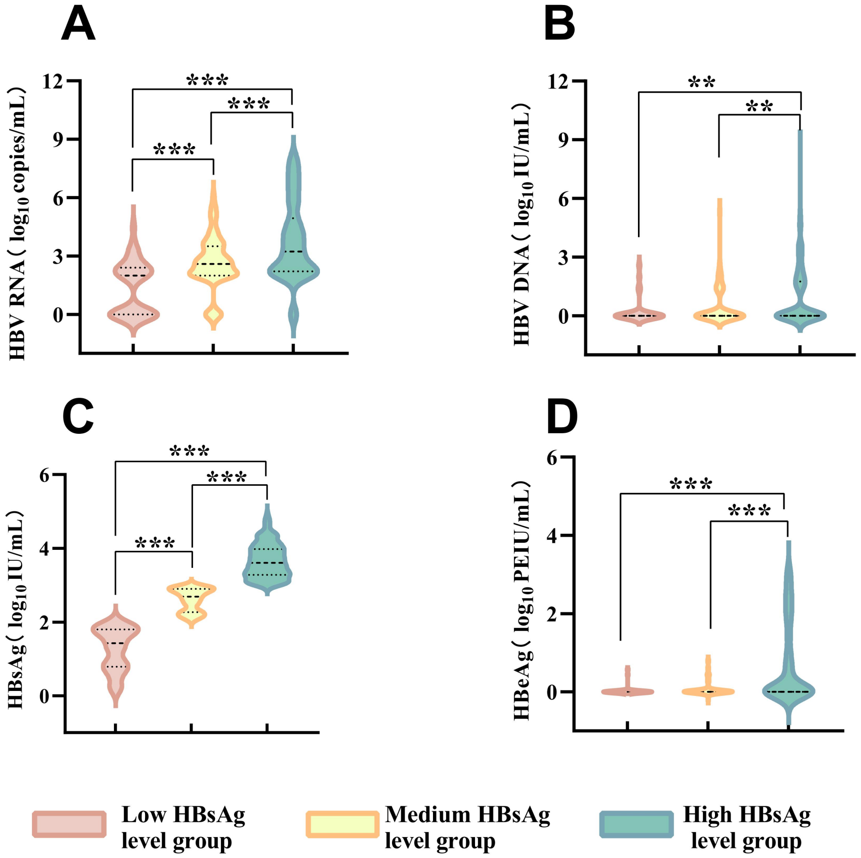

Fig. S2. Indicators with significant differences under HBsAg level grouping (A: HBV RNA; B: HBV DNA; C: HBsAg; D: HBeAg;). **, *P* <0.01; ***, *P* <0.001.

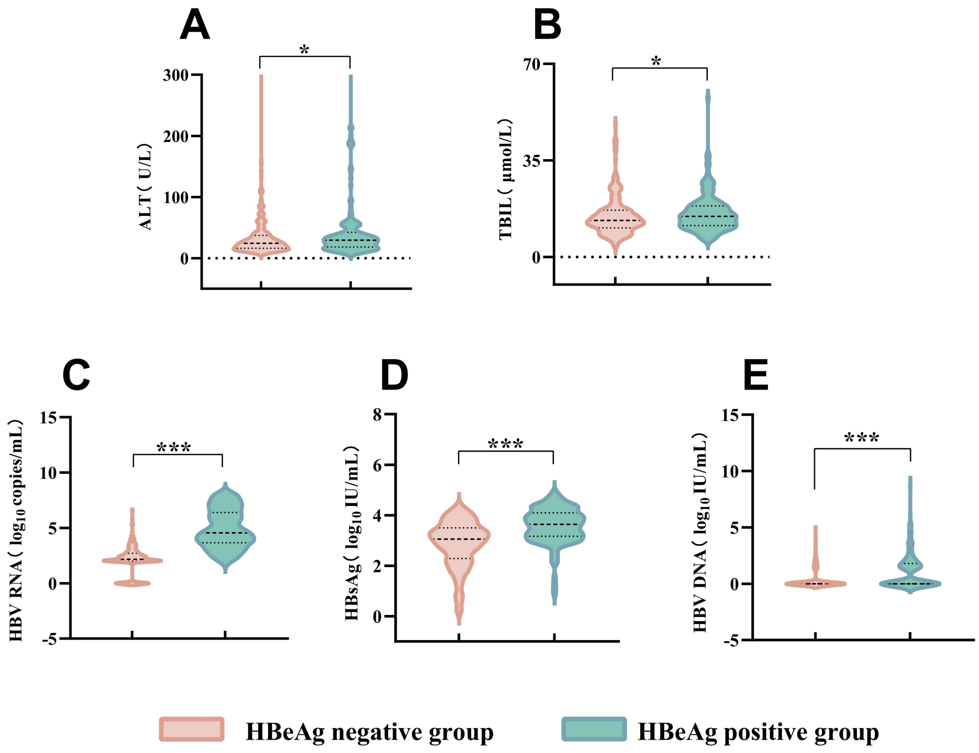

Fig. S3. Significant differences in indicators between the HBeAg-negative group and the HBeAg-positive group (A: ALT; B:TBIL; C HBV RNA; D: HBsAg; E: HBV DNA). *, P <0.05; ***, P <0.001.

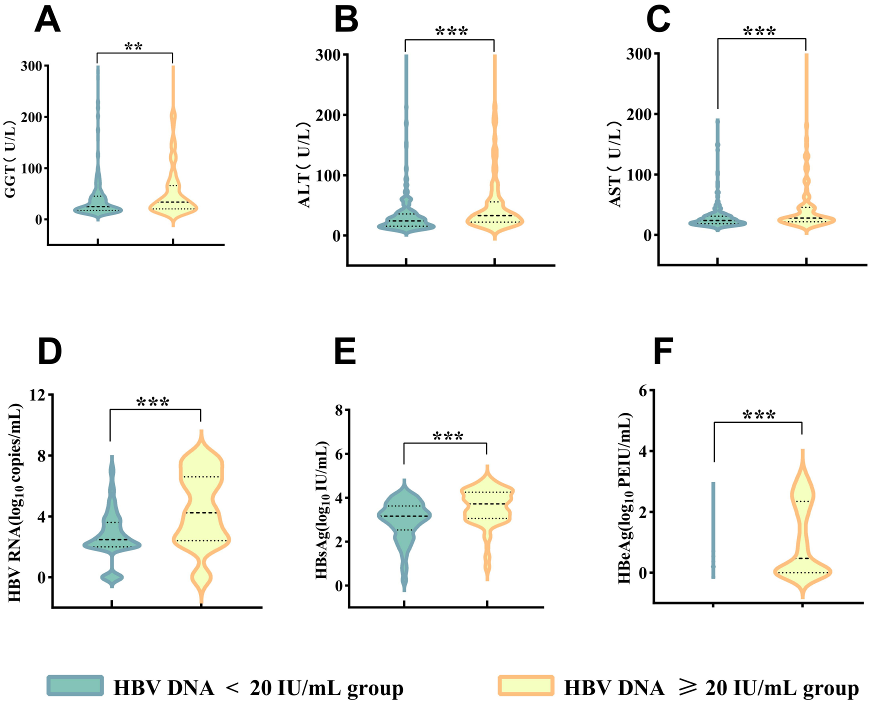

Fig. S4. Significant differences in indicators between the HBV DNA < 20 IU/mL group and the HBV DNA ⩾ 20 IU/mL group (A: GGT; B: ALT; C: AST; D: HBV RNA; E: HBsAg; F: HBeAg). **, P < 0.01; ***, P < 0.001.
